# Supplementary figures and images for: Differentially expressed serum proteins in children with or without asthma as determined using isobaric tags for relative and absolute quantitation proteomics
Source: PeerJ. 2020 Nov 3;8:e9971. doi: 10.7717/peerj.9971 (PMC7646293; doi:10.7717/peerj.9971)

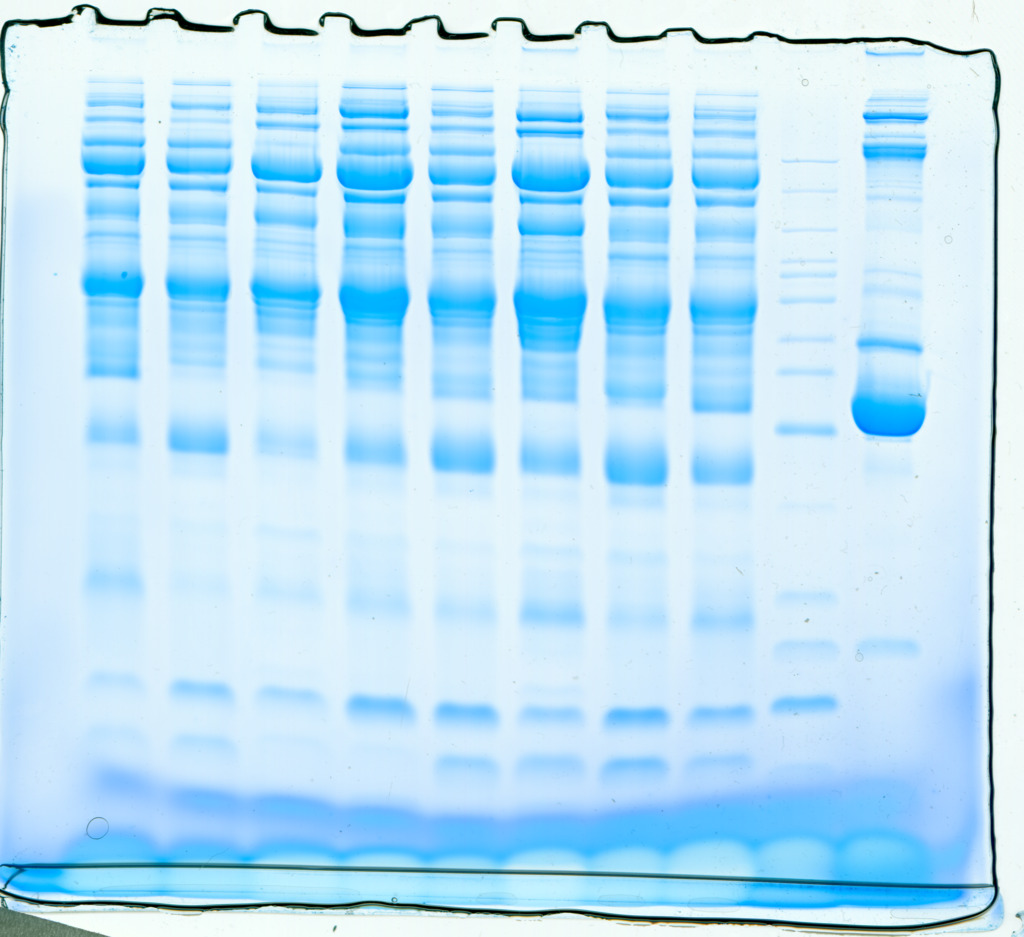

Supplement: Supplemental Information 1 [file peerj-08-9971-s001.jpg]
